# DNA methylation changes of whole blood cells in response to active smoking exposure in adults: a systematic review of DNA methylation studies

**DOI:** 10.1186/s13148-015-0148-3

**Published:** 2015-10-16

**Authors:** Xu Gao, Min Jia, Yan Zhang, Lutz Philipp Breitling, Hermann Brenner

**Affiliations:** Division of Clinical Epidemiology and Aging Research, German Cancer Research Center (DKFZ), Im Neuenheimer Feld 581, D-69120 Heidelberg, Germany; Division of Preventive Oncology, German Cancer Research Center (DKFZ) and National Center for Tumor Diseases (NCT), Im Neuenheimer Feld 460, D-69120 Heidelberg, Germany; German Cancer Consortium (DKTK), German Cancer Research Center (DKFZ), Im Neuenheimer Feld 280, D-69120 Heidelberg, Germany

**Keywords:** DNA methylation, Active smoking, Whole blood cells, Systematic review

## Abstract

Active smoking is a major preventable public health problem and an established critical factor for epigenetic modification. In this systematic review, we identified 17 studies addressing the association of active smoking exposure with methylation modifications in blood DNA, including 14 recent epigenome-wide association studies (EWASs) and 3 gene-specific methylation studies (GSMSs) on the gene regions identified by EWASs. Overall, 1460 smoking-associated CpG sites were identified in the EWASs, of which 62 sites were detected in multiple (≥3) studies. The three most frequently reported CpG sites (genes) in whole blood samples were cg05575921 (*AHRR*), cg03636183 (*F2RL3*), and cg19859270 (*GPR15*), followed by other loci within intergenic regions *2q37.1* and *6p21.33*. These significant smoking-related genes were further assessed by specific methylation assays in three GSMSs and reflected not only current but also lifetime or long-term exposure to active smoking. In conclusion, this review summarizes the evidences for the use of blood DNA methylation patterns as biomarkers of smoking exposure for research and clinical practice. In particular, it provides a reservoir for constructing a smoking exposure index score which could be used to more precisely quantify long-term smoking exposure and evaluate the risks of smoking-induced diseases.

**Electronic supplementary material:**

The online version of this article (doi:10.1186/s13148-015-0148-3) contains supplementary material, which is available to authorized users.

## Background

Tobacco smoking is a major public health problem, globally associated with substantial preventable morbidity [1]. In particular, active smoking in adults accounts for a large share of a wide spectrum of chronic diseases, including various forms of cancer, respiratory, and cardiovascular diseases. DNA methylation, one of the main forms of epigenetic modifications, has recently been suggested to play an important role in the pathways of smoking and smoking-induced diseases [2].

Earlier epigenetic studies on active smoking generally applied locus-specific assays to detect cytosine conversion through PCR or DNA sequencing techniques to investigate the alteration of genes in tissues and blood samples [3, 4]. These approaches yielded several replicable smoking-related loci in the candidate genes *MAOA*, *MAOB*, and *COMT*, as well as global methylation differences [4]. Following the introduction of Infinium Human Methylation Bead Chip (27 and 450 K) assays, numerous smoking-related CpG sites have been discovered via epigenome-wide association studies (EWASs), which investigated DNA methylation in whole blood samples collected through epidemiological studies. The first such site, cg03636183, located in the gene *F2RL3*, was reported by Breitling et al. in 2011 [5]. The so far largest EWAS by Zeilinger, reported in 2013, discovered and validated statistically significant differences in the methylation of 187 CpG sites between current smokers and never smokers [6]. Based upon the findings in EWASs, more recent studies focused on blood DNA methylation at specific genes, such as *F2RL3* and *AHRR*, to further explore long-term or lifetime correlates of active smoking exposure which might serve as biomarkers for both current and past smoking exposure [7–9].

In view that blood samples are most commonly available in epidemiological studies and may be particularly relevant for systemic sequelae of smoking and smoking-related health disorders, it is predictable that there will be many EWASs based on whole blood samples in the near future with the spreading application of Illumina assays. Thus, this systematic review aims to summarize the current evidence on the association of active smoking with whole blood DNA methylation among adults, from EWASs and gene-specific studies (GSMSs) on specific gene regions identified by EWASs, to provide applicable suggestions for further smoking-related methylation studies.

## Methods

We conducted this systematic literature review according to a predefined protocol. Reporting follows the PRISMA statements [10].

### Data sources and search strategy

A systematic literature search was conducted to identify studies assessing smoking-related differential methylation in CpG sites in humans. Databases of PubMed and ISI Web of Science were searched for eligible articles until February 28, 2015. The keyword combinations used in PubMed were as follows: (((“smoking”[MeSH Terms] OR “smoking”[All Fields]) OR (“smoke”[MeSH Terms] OR “smoke”[All Fields])) AND (“methylation”[MeSH Terms] OR “methylation”[All Fields])). The search terms used in ISI Web of Science were as follows: TS = (((smoking*) OR (smoke)) AND (methylation)). After filtering the duplicates by Endnote X7, and excluding ineligible articles by title and abstract review, articles with potential relevance for the study topic underwent full-text review.

### Study selection

**Fig. 1:**
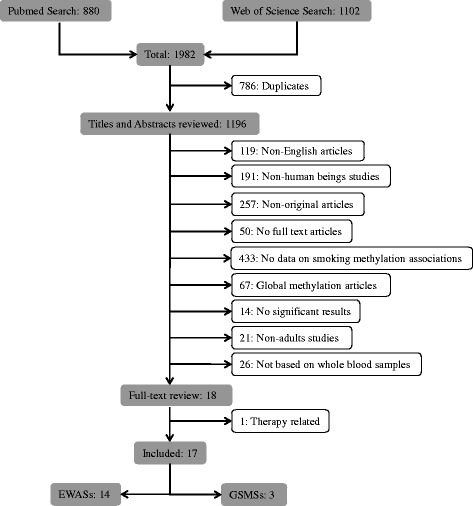
Flow diagram of systematic literature search in PubMed and Web of Science

During title and abstract screening for potentially eligible studies, we used the following exclusion criteria (Fig. 1): 1) not published in English; 2) studies conducted in non-human beings; 3) not original articles or articles without full text; 4) no data on smoking-methylation associations; 5) studies addressing global methylation only; 6) studies without any significant results; 7) studies not including adults; and 8) studies not based on blood samples. After a full-text review, therapy-related studies were also excluded, and the remaining articles were finally categorized into EWASs and GSMSs.

### Data extraction and quality assessment

Two investigators (XG, MJ) independently extracted data from the eligible studies. From each study, we summarized available information on first author, country, study design, basic characteristics of samples (including size, gender, and age distribution), type of bio-specimen, leukocyte distribution adjustment (LDA), smoking indicators, number of significant CpG sites along with their located genes, correction for multiple testing, and estimated effect sizes of smoking exposure on methylation (expressed by *β* values). Effect sizes, a quantitative measure of methylation changes, were calculated between current/former smokers and non-smokers for each CpG site without further discrimination by exposure quantity (effect size = median/mean *β*_current/former smoker_ – median/mean *β*_never smoker_).

We assessed the quality of each included article using the following quality criteria: 1) clear definition and description of study population; 2) suitable ascertainment of smoking and covariates (interviews/questionnaires or hospital records); and 3) consideration (through stratification or adjustment) of at least the following covariates in the analysis: age and sex.

Microsoft Excel 2010 (Microsoft Corporation, New Mexico, USA), SAS version 9.3 (SAS Institute Inc., Cary, NC, USA), and Sigmaplot Ver.12.5 (Systat Software Inc. California, USA) were used for data cleaning and to plot the figures, respectively.

## Results

### Literature search results

**Fig. 2:**
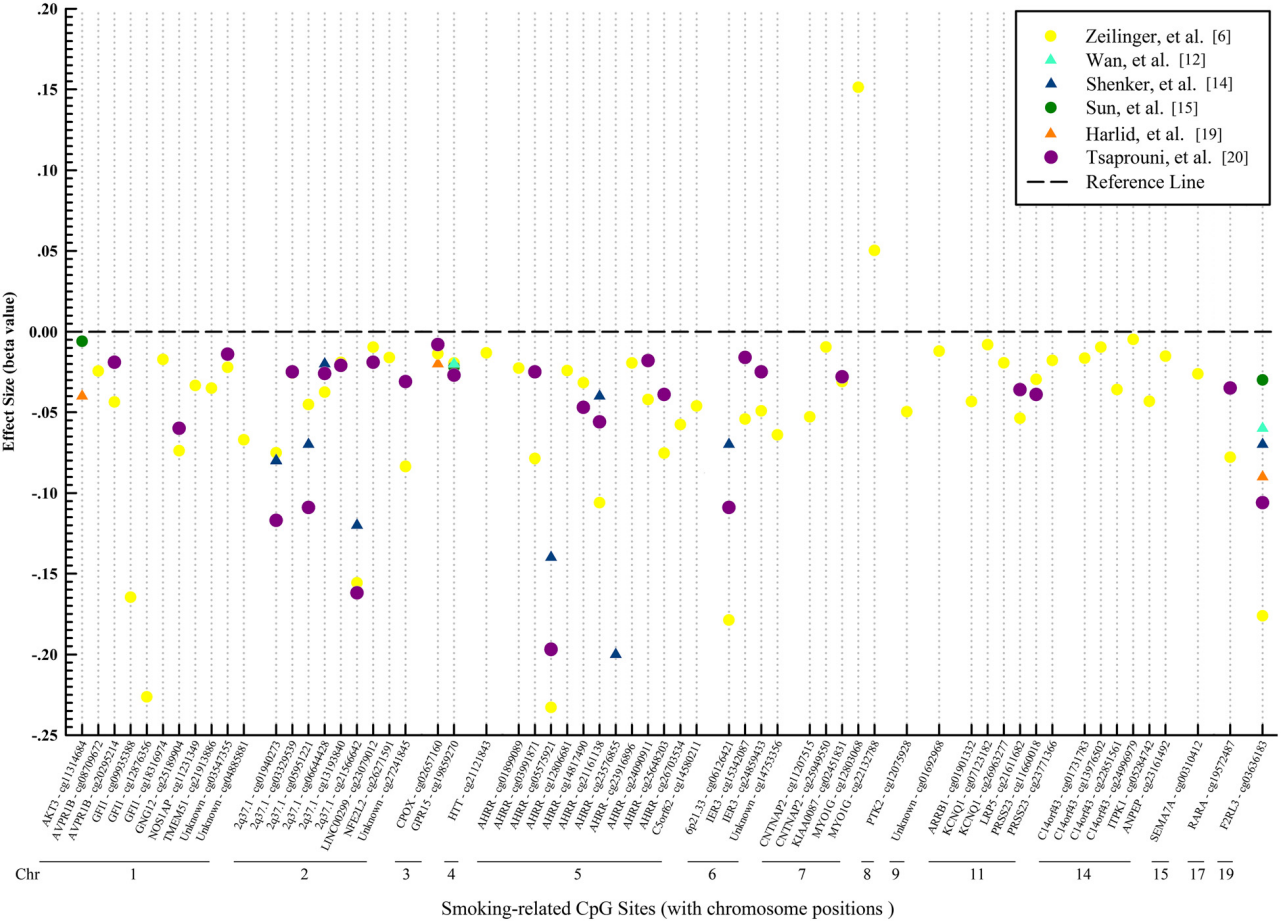
Effect sizes of smoking-related CpG sites reported ≥3 times (beta values of independent validation samples are shown where available). *Chr* chromosome position, *triangles* and *dots* represent the mean and median of beta values, respectively

**Fig. 3:**
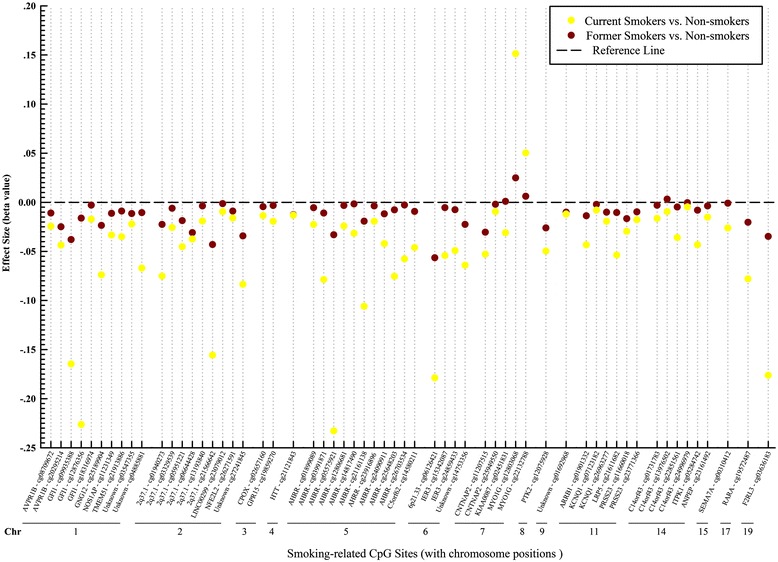
Effect sizes of smoking-induced methylation for current smokers and former smokers compared with non-smokers. *Chr* chromosome position, used the publically available data of Zeilinger et al. (median beta values) [6]

The initial search yielded 1982 articles using the above-described search terms, 880 from PubMed and 1102 from Web of Science (Fig. 1). Among those, we excluded 786 duplicates and 1178 by title and abstract scan. The remaining 18 studies were selected for a full-text review, of which one therapy-related study was excluded. Cross-referencing revealed no further relevant records. Therefore, 17 articles assessing the association of smoking with blood DNA methylation were eligible for this systematic review, including 14 EWASs [5, 6, 11–22] and 3 GSMSs [7, 8, 23] which were published between 2011 and 2015 (Tables 1 and 2). Furthermore, we summarized effect sizes of smoking exposure on all reported CpG sites with their corresponding genes (Additional file 1: Table S1) and plotted loci discovered by multiple (≥3) studies with their available effect sizes (*β* values) in validation panels (i.e., independent replication samples, which should not be affected by the overestimation of true associations that is to be expected in the discovery samples [24] Fig. 2). Additionally, we used the published data of the study by Zeilinger et al. as an example to describe the effect size alterations of multiple discovered loci after smoking cessation (Fig. 3) [6].

**Table 1:** Characteristics summary of EWASs based on whole blood samples

First author (year)	Country	Discovery sample			Validation sample			LDA^a^	Measurement	Correction for multiple tests^b^	Smoking indicators^c^	Identified number of CpGs (genes)^d^
		Size	Male	Mean age	Size	Male	Mean age					
		(*n*)	(%)	(year)	(*n*)	(%)	(year)					
Guida, (2015) [21]	UK	745	0	NA^e^	NA	NA	NA	Yes^f^	450 K	FWER	Current/former/never smoker (TQ)	461 (352)
Zaghlool, (2015)^h^ [22]	Qatar	123	35.8	37.9	–	–	–	Yes^f^	450 K	FDR	Smoker/non-smoker	8 (4)
Besingi, (2014)^h^ [16]	Sweden	432	45.8	42.7	–	–	–	Yes^f^	450 K	FDR	Smoker/ non-smoker	95 (66)
Dogan Mv Fau (2014)^i^ [17]	USA	111	0	48.4	62	0	49.0	Yes^f^	450 K/quantitative-PCR	FDR	Smoker/non-smoker (PY)	910 (625)
Elliott, (2014) [18]	UK	192	100	48.5	NA	NA	NA	Yes^f^	450 K	FWER	Current/former/never smoker (PY)	29 (15)
Harlid, (2014) [19]	USA	908	0	55.7	200/476^j^	0	53.3	No	27/450 K^k^	FDR	Current/former/never smoker (PY, TQ)	13 (13)
Tsaprouni, (2014) [20]	UK	464	70.5	55.4	356	0	60.1	Yes^f^	450 K	FWER	Current/former/never smoker	30 (15)
Shenker, (2013) [14]	UK	184	0	NA	190	71.6	NA	No	450 K/Pyrosequencing	FDR	Current/former/non -smoker (CL)	9 (4)
Sun, (2013) [15]	USA	972	29.3	66.3	239	28.5	40.8	Yes^f^	27/450 K^k^	FDR	Current/former/non -smoker (PY)	5 (5)
Philibert, (2013) [13]	USA	107	100	22.0	NA	NA	NA	No	450 K	FDR	Smoker/non-smoker (PY, CL)	3 (1)
Zeilinger, (2013) [6]	Germany	1793	51.3	60.9	478	48.3	51.8	Yes^g^	450 K	FWER	Regular/occasional/former/never smoker (ND, TQ, PY)	187 (94)
Philibert, (2012)^i^ [11]	USA	399	45.4	19.3	NA	NA	NA	No	450 K	FDR	Smoker/ non-smoker (PY)	1 (1)
Wan, (2012) [12]	USA	1085	54.4	57.3	369	35.8	47.5	No	27 K/Pyrosequencing	FDR	Current/former/never smoker (TQ, PY)	15 (14)
Breitling, (2011) [5]	Germany	177	50.8	54.0	316	59.5	55.0	No	27 K/MALDI-TOF^l^	FWER	Heavy/never/former smoker	1 (1)

^a^ *LDA* leukocyte distribution adjustment

^b^ *FWER* family-wise error rate, *FDR* false discovery rate

^c^ *PY* pack-year, *ND* numbers of cigarettes daily, *TQ* time of quitting smoking, *CL* serum cotinine levels

^d^Part of CpG sites located in the intergenic regions without gene annotations

^e^Not applicable

^f^The influence of leukocyte composition was adjusted using the algorithm described by Houseman et al. [25]

^g^The influence of leukocyte composition was adjusted by subtype cell count

^h^This study used the data published by other studies for replicating their significant sites

^i^The study was applied in the lymphocyte samples specifically

^j^Two-hundred samples were analyzed by 450 K, and 476 samples were analyzed by pyrosequencing

^k^The Illumina 27 and 450 K were used for exploratory and validation analysis, respectively

^l^MALDI-TOF = Sequenom matrix-assisted laser desorption ionization time-of-flight mass spectrometry

**Table 2:** Characteristics summary of GSMSs based on whole blood samples

First author (year)	Country	Sample			Measurement^a^	Smoking indicators^b^	Gene(s)	Main finding^e^
		Size	Male	Age mean				
		(*n*)	(%)	(year)				
Zhang, (2014) [8]	Germany	3588	44.4	61.7	MALDI-TOF	Current/former/never smoker (PY, ND, TQ)	*F2RL3*	*F2RL3* methylation strongly decreased with current smoking intensity and with lifetime cumulative smoking, and reached a plateau at higher current and cumulative smoking intensity. Methylation levels increased with time since cessation, but the time for full recovery was more than 20 years.
Shenker, (2013) [7]	UK	81/180^c^	81.5/0	NR^d^	BPP	Current/former/never smoker (SD, CL)	*AHRR*, *2q37*, *6p21*	Combining four gene loci of *AHRR*, *2q37*, and *6p21* into a single methylation index provided high positive, predictive, and sensitivity values for predicting former smoking status in both test (*n* = 81, AUC = 0.82) and validation (*n* = 180, AUC = 0.83) sample sets.
Breitling, (2012) [23]	Germany	1100	66.6	58.0	MALDI-TOF	Continuing smoker/quit after acute event/quit before acute event/never smoking	*F2RL3*	*F2RL3* were significantly associated with smoking history of patients with CHD (median methylation intensities at CpG_4, continuing smoker = 0.53; quit after acute event = 0.51; quit before acute event = 0.66; never smoking = 0.74) (*p* < 0.001).

^a^ *MALDI-TOF* Sequenom matrix-assisted laser desorption ionization time-of-flight mass spectrometry, *BPP* Bisulphite pyrosequencing

^b^ *PY* pack-year, *SD* smoking duration, *ND* numbers of cigarettes daily, *TQ* time of quitting smoking, *CL* serum cotinine levels

^c^The exploratory and validation analysis included 81 and 180 participants, respectively

^d^Not reported

^e^ *AUC* area under the curve, *CHD* coronary heart disease

### Study characteristics and results of EWASs

All EWASs applied Illumina 27 or 450 K methylation arrays to uncover smoking-related CpG sites. Three studies carried out pyrosequencing [12, 14, 19], one used MALDI-TOF [5], and one used quantitative-PCR [17] as additional supplementary measurement in their validation phases (Table 1). Six studies were from the USA, seven from Europe (UK = 4, Germany = 2, Sweden = 1), and one from Qatar. The population sizes varied from 107 to 1793 in discovery panels, two studies only included males [13, 18], and four only included females [14, 17, 19, 21]. Eight studies validated their findings further in internal independent replication samples, while another two studies replicated findings of significant associations from proceeding EWASs [16, 22]. Two studies were carried out in lymphocyte DNA [11, 17], and twelve studies assessed methylation in whole blood DNA. Eight studies adjusted their results for leucocyte distribution [6, 15–18, 20–22], seven of which used the algorithm of Houseman et al. [25]. However, five studies emphasized that the variability in DNA methylation due to cell composition was probably small and insignificant when compared with the differential methylation patterns that can be observed between smokers and non-smokers [15–17, 20]. Family-wise error rate (FWER) or false discovery rate (FDR) was applied to adjust multiple testing.

A total of 1460 CpG sites were identified in these EWASs, 62 of them were discovered in multiple (≥3) studies. Among the latter, 47 were located in 27 genes (35 were located in the bodies of corresponding genes), and 15 were located in the intergenic regions (Additional file 1: Table S1). Their effect sizes in validation samples are summarized in Additional file 1: Table S1 (range in validation samples, −0.233 (cg05575921) to +0.157 (cg23480021)), and compared in Fig. 2. The five sites (genes) with the largest effect sizes were cg09935388 (*GFI1*), cg05575921 (*AHRR*), cg23576855 (*AHRR*), cg06126421 (*6p21.33*), and cg03636183 (*F2RL3*). Loci cg03636183 (*F2RL3*), cg05575921 (*AHRR*), and cg19859270 (*GPR15*) were reported 12, 11, and 10 times, respectively. Other CpG sites located in intergenic regions of *2q37.1* and *6p21.33* were reported at least four times as well (Additional file 1: Table S1). For all but two sites within gene *MYO1G* (cg12803068 and cg22132788), smoking was associated with decreased methylation. Eleven loci discovered in multiple (≥3) studies were located in *AHRR*, followed by six loci in *2q37.1*, four loci in *C14orf43*, and three loci in *GFI1* (Fig. 2).

Figure 3 provides a comparison of effect estimates for these same CpG sites for current and former smokers from the largest EWAS up to date by Zeilinger et al. [6]. A consistent pattern of effects among former smokers that are in the same direction but much smaller than effects among current smokers suggests the reversibility of smoking-associated changes after cessation. This finding was further confirmed in two studies of Tsaprouni et al. and Guida et al. [20, 21]. They described patterns consistent with the reversal of changes in methylation intensity after smoking cessation and suggested the existence of dynamic, reversible site-specific methylation alterations due to active smoking. In addition, a study investigating both tobacco and snuff smoking showed that tobacco smoking, not smokeless tobacco, was involved with DNA methylation, indicating that the majority of methylation changes might be caused by the burnt products of tobacco, not its basic components [16]. Lastly, the studies of Elliott et al. and Zaghlool et al. identified ethnic heterogeneity of smoking-associated differential methylation patterns in several loci [18, 22]. For instance, smoking-associated differences of methylation at cg05575921 within *AHRR* were significantly lower in individuals of European origin compared to South Asians [18]. The two studies speculated that either key aspects of smoking behavior had not been captured or there was a true ethnic difference in methylation response to smoking exposure.

### Study characteristics and results of GSMSs

Four gene regions (*F2RL3*, *AHRR*, *2q37*, and *6p21*) and their related specific CpG sites, which had been identified by previous EWASs, were investigated in three whole blood-based GSMSs of active smoking (Table 2) [7, 8, 23]. All three studies were from Europe (Germany = 2, UK = 1) and population sizes varied from 261 to 3588. Two German studies evaluated the dose-response relationship between smoking exposure and methylation of *F2RL3* by MALDI-TOF. The earlier one also revealed strong associations of smoking with adverse outcomes of coronary heart disease and concluded that methylation in *F2RL3* might be a potential mediator of the detrimental impact of smoking [23]. The largest gene-specific study in 2014 was based on 3588 older adults from the general population [8]. This study disclosed clear dose-response relationships of *F2RL3* methylation with both current and lifetime smoking intensity, as well as with time since smoking cessation. In particular, it was demonstrated that it took more than 20 years for a full “recovery” of methylation levels. The study by Shenker et al. assessed genome regions *AHRR*, *2q37.1*, and *6p21.33* via pyrosequencing [7].

## Discussion

Active smoking is an established critical factor for epigenetic modifications in blood DNA [4]. To our knowledge, this is the first systematic literature review on this topic. We identified 17 eligible articles, which explored the association of active smoking exposure with epigenetic changes in blood DNA. Overall, 1460 smoking-related CpG sites were identified in 14 EWASs, 62 of which were discovered by multiple (≥3) studies. The most frequently reported sites were cg05575921 (*AHRR*), cg03636183 (*F2RL3*), cg19859270 (*GPR15*), and other loci within the intergenic regions *2q37.1* and *6p21.33*. Prominent findings for these smoking-related genes were further analyzed in GSMSs to disclose dose-response relationships of smoking intensities and time since cessation with methylation levels. Taken together, these studies suggest the possibility of using methylation markers for a refined quantification of smoking exposure and to better predict the risks of smoking-related diseases.

Smoking-induced methylation could occur in many regions of the human genome. In the annotated gene regions, approximately half of the smoking-related CpG sites are located in the body of specific genes (e.g., *AHRR*, *F2RL3*, etc.). Notably, their effect size was commonly higher than that of sites located at other parts, including the 1st exon, untranslated region (UTR), and transcription start site (TSS) (Additional file 1: Table S1, Fig. 2). Additionally, about one quarter of smoking-related loci are located in the intergenic regions, such as *2q37.1* and *6p21.3* (Additional file 1: Table S1).

For the sites located in the gene bodies, epidemiological studies have meanwhile observed biologically plausible associations with smoking-induced chronic diseases or cancers. For instance, the first discovered smoking-associated site cg03636183 is located in the body of gene *F2RL3* (the coagulation factor II receptor-like 3 gene) [5], and was consistently confirmed in multiple EWASs and even replicated across racial groups [15, 17–19, 22]. The function of *F2RL3* is coding the thrombin protease-activated receptor-4 (PAR-4), which is a protein expressed in various tissues over the body, including blood leukocytes and lung tissue, and plays a key role in platelet activation and cell signaling. This could partly explain why the methylation pattern of *F2RL3* was found to be related to risks for cardiovascular diseases (CVD) and lung cancer, as well as to total mortality [26, 27]. Nevertheless, the role of *F2RL3* as a potential mediator or just an indicator of smoking-related risk is still not well understood.

The strongest and most consistent associations have meanwhile been reported for CpG sites located in the body of *AHRR*, a well-known tumor suppressor. Smoking could trigger the generation of polycyclic aromatic hydrocarbons (PAHs) that affects the aryl hydrocarbon receptor (AHR), leading to alterations in the expression (and methylation status) of *AHRR* [3]. Thus, this gene could mediate detoxification of PAHs and might be involved in the metabolism of endogenous toxins from cigarette smoking [28]. A recent study by Zhang et al. disclosed clear dose-response relationships of *AHRR* methylation with both current and lifetime smoking exposure, as well as with smoking-related mortality outcomes [29]. Methylation at cg05575921in *AHRR* and a locus in *6p21.33* were additionally suggested to be promising candidates for enhancing cardiovascular risk prediction [29].

Irrespective of full understanding of the pathophysiological mechanisms, strong and consistent associations with smoking of a variety of CpG sites suggest their potential use as main correlates for smoking exposure. Two GSMSs have demonstrated the potential of several sites within *F2RL3* as promising biomarkers for both current and past smoking exposure [8, 23]. A recent study has identified that cg05575921 within *AHRR* was both sensitive and specific for current smoking in adults with an area under the curve (AUC) of 0.99, and efforts evaluating methylation of cg05575921 as a biomarker to guide smoking cessation are ongoing [30]. Further studies on loci within more smoking-related genes are in need to explore precise dose-response relationships to describe smoking exposure globally and understand their molecular mechanisms comprehensively.

In addition to the critical sites in gene bodies, there are several smoking-related sites within other genome regions, such as cg19859270, which are located at the 1stExon of gene *GPR15* (G-protein-coupled receptor 15), and several loci in the intergenic region *2q37.1*. These sites might make additional contributions to smoking exposure evaluation through a smoking-related methylation signature. Along with the significant loci in *AHRR* and *F2RL3*, they could facilitate the construction of a quantitative approach with better specificity to differentiate never smokers from former smokers (validated AUC = 0.83, positive predictive value = 0.85) [7]. In 2012, *GPR15* was first reported in the study of Wan et al., along with its relationships with current and long-term smoking [12]. Afterwards, the study of Tsaprouni et al. presented that this gene was the only one showing a clear trend of increased gene expression in smokers compared to non-smokers with a prominent negative correlation between gene expression and methylation [20]. The author thus presumed that the reduction of methylation levels of locus cg19859270 within *GPR15* in smokers would lead to increased transcription. This differential expression of *GPR15* in smokers compared with never smokers was further confirmed by recent studies of Bauer et al. and Kõks et al. [31, 32]. In addition, as an HIV co-receptor, this gene was recently reported to interact with the ethnicity-dependent differential prevalence of HIV, especially HIV2 in African Americans [33]. Moreover, the six significant sites in locus *2q37.1* are directly located adjacent to a cluster of alkaline phosphatase genes [34].

In the implementation and interpretation of EWASs and GSMSs based on blood samples, a potential limitation deserving particular attention is that whole blood DNA represents a mixture of DNA from various types of leucocytes that show partly different methylation patterns. Hence, smoking-related differential methylation may, in theory, partly reflect smoking-related shifts in leucocyte distribution. The majority of EWASs adjusted their analysis for leucocyte distribution with the algorithm of Houseman et al. [25]. However, although smoking is known to increase the overall numbers of leucocytes [35], the impact on leucocyte distribution still remains unclear [15–17, 20, 21]. Recently, *GPR15* methylation has been shown to be linked with chronic inflammation via regulating T cell migration [32], which raised the possibility that for some loci, like *GPR15*, differential methylation might reflect a shift in blood cell mixture. On the other hand, a recent study compared the smoking-related methylation profiles in both buccal and whole blood samples and found that effect sizes in blood samples were similar to that in buccal samples [36]. This suggests that cell type distribution has no major impact for the majority of smoking-related differential methylation. Nevertheless, even if smoking-related methylation patterns were partly due to confounding by leucocyte distribution, they might still be useful as biomarkers for smoking exposure.

Our review was limited to smoking-associated DNA methylation changes among adults. Similar to the findings in adults, EWASs investigating the role of maternal smoking in newborns also identified differentially methylated CpG sites in several smoking-related genes, such as *AHRR*, *MYO1G*, and *GFI1*, but with less pronounced effect sizes. Interestingly, several loci, such as cg23067299 (*AHRR*) and cg05549655 (*CYP1A1*), were, so far, only discovered in studies assessing the impact of maternal smoking in newborns, whereas none of these studies reported differential methylation of cg03636183 (*F2RL3*) and cg19859270 (*GPR15*), two critical loci associated with adult smoking. These discrepancies are likely to be explained by the differences of exposure pathways and population susceptibilities [3, 37], but more and larger EWASs in the respective age groups are needed to further clarify similarities and differences.

The number of known smoking-related CpG sites continues to increase. Given that smoking is an established risk factor for many chronic diseases, these loci could have important applications as objective biomarkers of both current and lifetime smoking exposure and for quantifying risks of smoking-related diseases. Recent GSMSs have already demonstrated strong dose-response relationships between methylation signatures and current and lifetime smoking exposure, as well as time since cessation of smoking [7, 8]. Furthermore, strong associations have been demonstrated between methylation signatures and a variety of major disease endpoints, including coronary artery diseases, lung cancer, and asthma [29, 38–41]. With further refinement of methylation signatures and further evaluation of the predictive value for these and additional disease outcomes, smoking-related methylation signatures might become a valuable tool for enhanced risk stratification and risk-adopted screening and treatment decisions in clinical practice. As a promising example, Teschendorff et al. constructed a smoking index score and subsequently showed that it was able to discriminate normal tissue from cancer tissue rather well [36], thereby demonstrating that smoking-related methylation indices could be useful risk indicators of smoking-induced health disorders.

This review has specific strengths and limitations. Strengths include the comprehensive search in two main databases, as well as strict adherence to standards of study selection, classification, and reporting. However, despite this comprehensive search strategy, we cannot exclude the possibility of having missed relevant studies, especially studies reported in languages other than English, or without full-length reports. Second, in most studies, smoking exposure was exclusively ascertained by self-reporting which is known to be less than perfect and most likely led to underestimation of true effects. Finally, our review was restricted to methylation patterns in blood DNA associated with active smoking. The focus on this sample matrix was a conscious decision due to its special relevance and ubiquitous availability in large epidemiological as well as routine point-of-care settings. Future research should address specific smoking-associated methylation signatures in various types of tissues (e.g., tumor samples, buccal cells). Also, apart from active smoking, methylation signatures reflecting passive smoking would be of major interest.

## Conclusions

In summary, since the first discovery of smoking-related differential DNA methylation by Breitling et al. in 2011 [5], methylation of a large number of CpG sites has been consistently shown to be affected by smoking exposure. With few exceptions, active smoking was associated with reduced methylation levels, and smoking-related changes in methylation turned out to be reversible, with intermediate methylation levels among former smokers compared to current and never smokers. However, several studies have shown that it may take up to 20 years to reach a full “methylation recovery” [8, 21]. Even though whole blood DNA presents a mixture of leucocytes subtypes, methylation of DNA from whole blood samples seems to be a powerful and highly informative biomarker not only for current smoking but also for lifetime history of smoking. Preliminary data suggest that smoking-related methylation signatures may also be very useful predictors of smoking-associated risks. Further research should aim for refinement of smoking-related methylation signatures and for their comprehensive validation with respect to a variety of disease outcomes in large epidemiological studies in order to best define the use of such signatures for research and clinical practice.

## Additional file

Additional file 1: Table S1. Details of CpG sites published in all active smoking EWASs included in the review. This table includes 5 sheets: 1) the general information of reported CpG sites in each included manuscript; 2) CpG sites reported ≥2 times; 3) characteristics of multiple reported (≥3 times) CpG sites; 4) the list of active smoking-related CpG sites (no duplicates); and 5) data of Fig. 2. (XLSX 284 kb)
